# Particle Swarm Optimization of Multilayer Multi-Sized Metamaterial Absorber for Long-Wave Infrared Polarimetric Imaging

**DOI:** 10.3390/mi15030319

**Published:** 2024-02-25

**Authors:** Junyu Li, Jinzhao Li, Fei Yi

**Affiliations:** 1 School of Optical and Electronic Information, Huazhong University of Science and Technology, Wuhan 430074, China; junyu.li@raytrontek.com (J.L.);; 2 IRay Technology Co., Ltd., Yantai 264006, China; 3 Wuhan National Research Center for Optoelectronics (WNLO), Huazhong University of Science and Technology, Wuhan 430074, China; 4 Optics Valley Laboratory, Wuhan 430074, China; 5 Shenzhen Huazhong University of Science and Technology Research Institute, Shenzhen 518000, China

**Keywords:** metamaterial absorber, infrared polarimetric imaging, particle swarm optimization

## Abstract

Infrared polarization imaging holds significant promise for enhancing target recognition in both civil and defense applications. The Division of Focal Plane (DoFP) scheme has emerged as a leading technology in the field of infrared polarization imaging due to its compact design and absence of moving parts. However, traditional DoFP solutions primarily rely on micro-polarizer arrays, necessitating precise alignment with the focal plane array and leading to challenges in alignment and the introduction of optical crosstalk. Recent research has sought to augment the performance of infrared detectors and enable polarization and spectral selection by integrating metamaterial absorbers with the pixels of the detector. Nevertheless, the results reported so far exhibit shortcomings, including low polarization absorption rates and inadequate polarization extinction ratios. Furthermore, there is a need for a comprehensive figure of merit to systematically assess the performance of polarization-selective thermal detectors. In this study, we employ the particle swarm optimization algorithm to present a multilayer, multi-sized metamaterial absorber capable of achieving a remarkable polarization-selective absorption rate of up to 87.2% across the 8–14 μm spectral range. Moreover, we attain a polarization extinction ratio of 38.51. To elucidate and predict the resonant wavelengths of the structure, we propose a modified equivalent circuit model. Our analysis employs optical impedance matching to unveil the underlying mechanisms responsible for the high absorption. We also introduce a comprehensive figure of merit to assess the efficacy of infrared polarization detection through the integration of metamaterials with microbolometers. Finally, drawing on the proposed figure of merit, we suggest future directions for improving integrated metamaterial absorber designs, with the potential to advance practical mid-infrared polarization imaging technologies.

## 1. Introduction

Polarization states describe the trajectory undertaken by the electric field vector of light. As one of the fundamental parameters of light, polarization states and their measurement are of great interest in almost all areas of optics [1,2,3]. Conventional thermal imagers measure the intensity of optical radiation across the entire infrared spectrum of interest. Often, the polarization signature of an artificial target will differ from that of the background, even when the target is in thermal equilibrium [4,5]. Consequently, infrared imaging polarimetry has the potential to expand target acquisition capabilities beyond what conventional thermal cameras can achieve. Diverse configurations exist in the field of infrared polarization imaging, with the Division of Focal Plane (DoFP) polarimeter playing an increasingly central role in the field due to its significantly reduced size, weight, and power (SWaP) requirements. Typical infrared DoFP polarimeters are realized by positioning a micro-polarizer array (MPA) in front of the focal plane array. The MPA-based imaging polarimeter for the 3–5 μm spectral band was demonstrated in 1999 for the first time [6]. In 2016, Polaris Sensor Technologies introduced the Pyxis imager, an infrared imaging polarimeter employing the DoFP configuration [7]. In these works, the micro-polarizers are fabricated separately and subsequently assembled with the focal plane array. This architecture is complicated, expensive, and incompatible with complementary metal–oxide–semiconductor (CMOS) technology. Furthermore, due to the inevitable presence of an air gap between the micro-polarizer and the detector pixel, the MPA-based architecture suffered from optical crosstalk issues among adjacent pixels and a diminished polarization extinction ratio under oblique incidence [8,9,10].

Recently, we introduced an innovative polarization imaging framework built upon a metamaterial absorber (MA) integration strategy, which directly combines nanostrip antenna-based MAs with microbolometer pixels [11,12,13]. Within this framework, carefully optimized MAs with spectral and polarization selectivity efficiently convert incident infrared radiation, which contains specific polarization information, into heat and conduct it to the microbolometer pixels. This approach diverges from the MPA-based architecture by eliminating the air gap between the integrated MAs and the microbolometer pixels, leading to a substantial reduction in optical crosstalk and an enhancement of the polarization extinction ratio. More importantly, the MA-based architecture goes beyond the MPA-based counterpart by allowing for the simultaneous resolution of spectral bands and polarization states. In contrast, MPAs are limited to the broad spectrum selection of polarization states. However, the MAs reported so far fall short in terms of absorption bandwidth and polarization selectivity, particularly in the longwave infrared (LWIR) band (as detailed in Table 1) [14,15]. In addition, a comprehensive figure of merit for evaluating various MA integrations with microbolometers for polarization imaging detection has yet to be established.

In light of these considerations, this study presents a polarization-selective MA designed for operation within the 8–14 μm band, based on a multilayered metal–insulator–metal–insulator–metal (MIMIM) architecture. An averaged absorption of 87.2% for the TM polarization and a polarization extinction ratio (PER) of 38.51 are achieved in the LWIR band by optimizing the MIMIM structure using the particle swarm optimization (PSO) algorithm [19], a robust and easy-to-implement algorithm. A modified equivalent circuit model and optical impedance matching analysis are used to reveal the underlying mechanism driving this highly polarization-selective absorption. By imposing additional constraints on the parameter range during the PSO optimization process, in accordance with fabrication limitations and the selection of CMOS-compatible materials, we have ensured the feasibility of manufacturing the designed absorber on a standard integrated circuit production line employing I-line steppers. Additionally, we introduce a comprehensive figure of merit to evaluate the performance of infrared polarization detection via the integration of MAs with microbolometers, and we offer a comparative assessment between the MIMIM absorber and the MIM absorber. Finally, we discuss the selection of metallic and dielectric materials suitable for the construction of the MA.

## 2. Results and Discussion

It has been reported that the conventional MIM structure with single-sized nanostrip antennas exhibits an absorption peak with limited bandwidth, and the resonant wavelength is proportional to the width of the nanostrip [20,21]. To broaden the absorption band, one approach is to incorporate multiple resonators operating at distinct wavelengths within a single unit cell [22]. These multi-sized resonators can be arranged in parallel, a configuration termed horizontal integration. However, the enhancement of bandwidth achieved through this integration method is hampered by the limited absorption cross-section of each individual resonator. Alternatively, another approach involves stacking the multi-sized resonators vertically, referred to as vertical integration. While vertical integration does not impose restrictions on the number of integrated resonators, it introduces a trade-off between thickness and absorption bandwidth. Increased thickness results in a larger thermal mass, thereby constraining the response speed of thermal detectors.

Here, we combine the strengths of both integration methods by transitioning from the conventional MIM structure to a more intricate MIMIM structure, thereby establishing a novel hybrid integration approach. Table 1 compares the proposed hybrid integration absorber with other reported absorbers with high polarization-selective absorption in LWIR [12,16,17,18]. On one hand, our proposed absorber achieves a large polarization extinction ratio and a high absorption of transverse magnetic (TM) polarization over a broad bandwidth. On the other hand, it is imperative to acknowledge that the MIMIM structure necessitates a greater thickness to attain its optimal performance, in contrast to the MIM structure. Later, we will carefully analyze the impact of the polarization extinction ratio, the absorbance of TM polarization, and the absorber’s thickness on the polarization detection.

**Figure 1:**
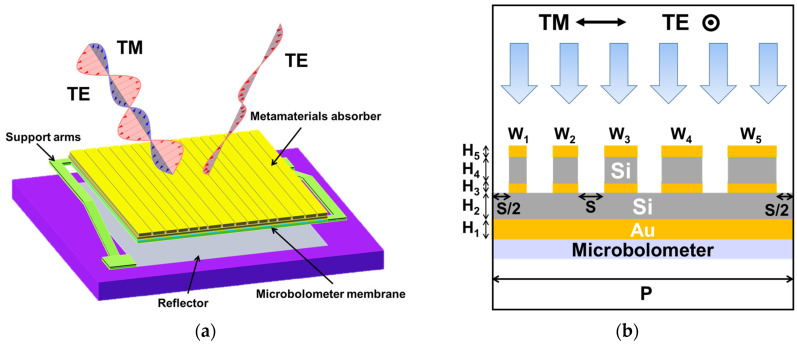
(**a**) Concept of polarization-selective MA-integrated microbolometer. (**b**) Schematic diagram of the proposed MIMIM absorber.

Figure 1a shows the concept of polarization-selective multilayer multi-Sized MA-integrated microbolometer, while Figure 1b shows the periodic unit of the MIMIM structure consisting of a gold backplate, a continuous silicon spacer layer, and a discrete silicon dielectric layer sandwiched between two metal nanostrip layers. The layer adjacent to the continuous dielectric layer is referred to as the first-layer nanostrips, while the topmost layer is designated as the second-layer nanostrips. This structural asymmetry endows the MIMIM structure with the capability to selectively absorb TM polarization while reflecting transverse electric (TE) polarization from the incident light. The absorbed optical energy subsequently undergoes conversion into heat through free carrier absorption within the metallic nanostructures, consequently raising the temperature of the microbolometers beneath [13,23]. For the sake of simplicity, we refer to the MIM absorber as the single-layer absorber and the MIMIM absorber as the double-layer or bilayer absorber.

The polarization states of light, including partially polarized light or unpolarized light, can be characterized by Stokes vectors [24]. These vectors, consisting of four real-valued elements, serve as a descriptive framework for elucidating the optical properties of partially polarized light. The Stokes vector essentially represents the combination of various measured light properties:

(1) $$S=\left|\begin{array}{c} \begin{array}{c} S_{0}\\ S_{1} \end{array}\\ \begin{array}{c} S_{2}\\ S_{3} \end{array} \end{array}\right|=\left|\begin{array}{c} \begin{array}{c} I_{0}+I_{90}\\ I_{0}-I_{90} \end{array}\\ \begin{array}{c} I_{45}-I_{135}\\ I_{RCP}+I_{LCP} \end{array} \end{array}\right|$$

where *I*_0_, *I*_45_, *I*_90_, and *I*_135_ represent the intensity of the linearly polarized components in the directions of 0°, 45°, 90°, and 135°, respectively. *I_RCP_* and *I_LCP_* correspond to the intensities of the and left-hand circular polarized components, respectively. For natural objects, it is typically acceptable to disregard the circular polarization component (*S*_3_ = 0) [25]. The polarization state of incident light can then be described using the degree of linear polarization (*DoLP*) and the angle of linear polarization (ϕ):

(2) $$DoLP=\frac{\sqrt{S_{1}^{2}+S_{2}^{2}}}{S_{0}}$$

(3) $$\phi =\frac{1}{2}tan^{-1}\left(\frac{S_{2}}{S_{1}}\right)$$

It then follows that the Stokes vectors associated with linear polarization, namely *S*_1_ and S_2_, can be expressed in terms of *S*_0_ as follows:

(4) $$S_{1}=S_{0}\cdot DoLP\cdot cos\left(2\phi \right)\;S_{2}=S_{0}\cdot DoLP\cdot sin\left(2\phi \right)$$

The performance of a polarizer can be characterized through diattenuation *D*, a parameter that is derived from the polarization extinction ratio (*PER*) [8]:

(5) D=(PER−1)/(PER+1)

For the proposed absorber, the *PER* can be defined as the average spectral absorption of TM polarization divided by the average spectral absorption of TE polarization in the 8–14 μm band:

(6) $$PER=\frac{A_{TM}}{A_{TE}}{|}_{8-14\mu m}$$

where *A_TM_* and *A_TE_* represent the spectral absorption of the TM polarization and TE polarization, respectively.

The critical system parameter to characterize a polarimeter’s performance is the noise-equivalent degree of linear polarization or *NeDoLP* [8]. The *NeDoLP* is defined as the root-mean-squared (RMS) noise of the measured *DoLP* when imaging a uniformly unpolarized scene, and this value is averaged across all operational pixels [7]. This metric provides insights into the polarimeter’s capability to detect different levels of polarization signals. The *NeDoLP* is related to the noise-equivalent temperature difference (*NETD*) via the uncertainty propagation equation [26], and the resulting *NeDoLP* is computed as follows:

(7) $$NeDoLP=\left(1+D\right)\sqrt{\frac{1}{2\cdot D^{2}}+\frac{DoLP^{2}}{4}}\cdot \frac{NETD}{T_{B}}$$

where *D* represents the diattenuation, *DoLP* stands for the degree of linear polarization of the injected signal, *NETD* signifies the noise-equivalent temperature difference of the sensor in Kelvin, and *T_B_* denotes the brightness temperature of the measured signal. According to this formula, when the *NETD* of the detector remains constant, there is limited performance improvement once the polarization extinction ratio exceeds 10 to 20 [8], a conclusion corroborated by various studies in the literature [27,28]. On the other hand, when *D* and *DoLP* are held constant, Equation (7) reveals that *NeDoLP* is proportional to the *NETD*. In 2011, Raytheon Vision Systems conducted experiments in LWIR polarimetry, investigating the relationship between *NETD* and NeDoLP, with the results confirming this deduction [27]. Therefore, when the polarization extinction ratio surpasses 20, reducing the *NETD* assumes greater significance than further augmenting the polarization extinction ratio. Given that the polarization extinction ratio of the proposed absorber can readily exceed 20 due to the substantial reflectivity of TE polarization, optimizing TM absorption emerges as a more pivotal objective as it subsequently reduces the *NETD*.

To optimize the TM absorption of the absorber, we employ a solver based on the finite-difference time-domain (FDTD) method to simulate the spectral absorption of the MIMIM structure. Additionally, we implement a particle swarm optimization (PSO) program to iteratively search for the optimal parameters governing the structure [11]. As depicted in Figure 1b, the thickness of the backplate (*H*_1_) is set as 100 nm, while the thicknesses of nanostrips (*H*_3_, *H*_5_) are set as 50 nm. Five parameters undergo optimization, which include the spacing (*S*) between adjacent strips, the tuning difference (Δ = *W_N_*_+1_ − *W_N_*, *N* = 1, 2, 3…), the thickness of the continuous dielectric spacer layer (*H*_2_), the average width of the nanostrips (*W*), and the thickness of the discrete dielectric spacer layer (*H*_4_). These parameters are organized into a vector *X* = [*S*, Δ, *H*_2_, *W*, *H*_4_], referred to as a particle. Initially, the PSO program generates several random particles. Subsequently, the FDTD solver conducts simulations of the spectral absorption for TM polarization and calculates the fitness function (*FF*) of each particle over the wavelength range spanning from 8 μm to 14 μm. Since optimizing the absorption rate of TM polarization is critical, the fitness function is defined as follows:

(8) $$FF=\int_{8\mu m}^{14\mu m}A_{TM}\left(\lambda \right)d\lambda$$

Subsequently, the particle undergoes an iterative exploration of the parameter space with the objective of maximizing the fitness function. In this work, the number *N* of particles in the swarm is 20 and the number of iterations is 50. This iterative process continues until the predetermined total number of iterations is attained, at which point the best-performing particle is identified as the optimal solution. For more details for the optimization process, please refer to reference [11]. For a more comprehensive understanding of the PSO optimization algorithm, additional details can be found in reference [29].

**Figure 2:**
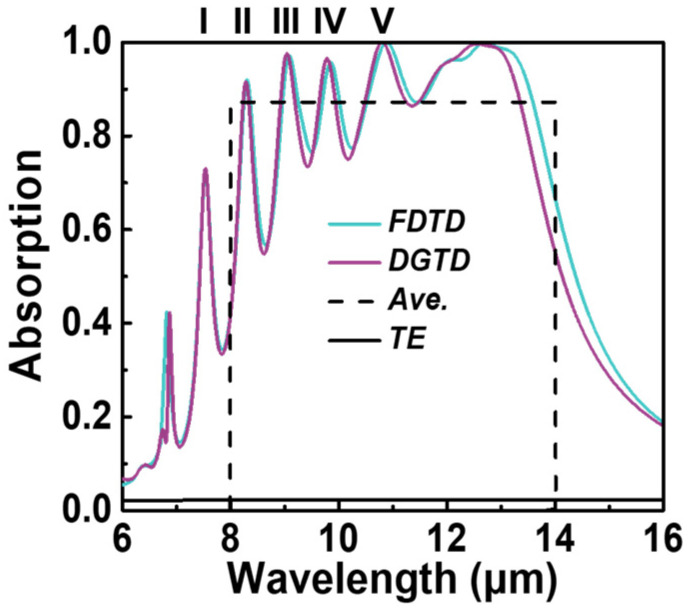
The optimized spectral absorption of the MIMIM structure under normal incidence.

In Figure 2, we present the polarization-selective absorption spectra of an optimized bilayer metamaterial absorber under normal incidence. In this configuration, we incorporate five nanostrips within a unit cell of the periodic structure, employing amorphous silicon as the spacing material. The refractive index of silicon is obtained from [30]. The computationally optimized spectral absorption of the TM and TE polarization are shown by the solid green and black lines, respectively. We have labeled the first five resonant wavelengths, arising from localized surface plasmon resonances (LSPRs), as I, II, III, IV, and V. It is worth noting that the resonant wavelengths from 12 μm to 13.5 μm are closely coupled and thus challenging to distinguish. Additionally, the narrowband resonance at approximately 7 μm is attributed to the excitation of surface plasmon polaritons (SPPs) [11]. The TM absorption rate averaged over the 8–14 μm band is denoted by the black dotted line, amounting to 87.2%, with a corresponding polarization extinction ratio of 38.51. To validate the outcomes obtained from the FDTD solver, we employ the Discontinuous Galerkin Time-Domain (DGTD) solver to compute the spectral absorption of the optimized structure. The results from the DGTD solver are represented by the purple line in Figure 2. The DGTD algorithm tackles the macroscopic Maxwell equations within a time-domain framework, relying on an unstructured simplex mesh [31]. It is evident that the absorption spectra obtained from both solvers exhibit good agreement.

**Figure 3:**
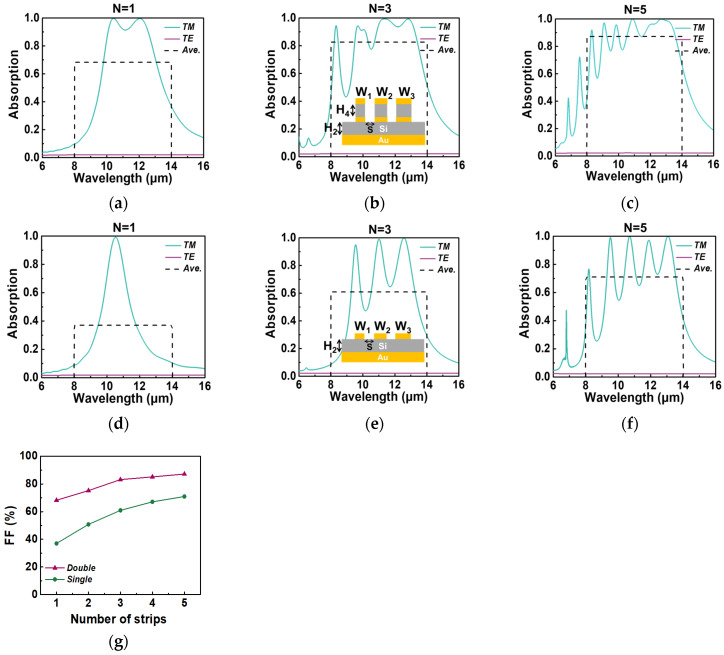
The spectral absorption of the TM polarization (green line) and TE polarization (purple line) for (**a**–**c**) optimized MIMIM design and (**d**–**f**) optimized MIM design, assuming the number of nanostrips per period is 1, 3, and 5, respectively. (**g**) The fitness function as a function of the number of nanostrips per period.

We proceed to investigate the impact of the number (*N*) of nanostrips per period on the optimized spectral absorption. We optimize the absorption spectra for *N* = 1–5 using the aforementioned method. The optimized absorption spectra for *N* = 1, 3, and 5 are displayed in Figure 3. Specifically, Figure 3a, Figure 3b and Figure 3c present the optimization results for the double-layer absorber with *N* = 1, 3, and 5, respectively, while Figure 3d–f depict the optimization outcomes for the single-layer absorber with the same values of *N*. The relevant geometric parameters of the optimized single-layer absorber and double-layer absorber are shown in Table 2. To illustrate the trends, we plot the fitness function, representing the spectrally averaged TM polarization absorption, as a function of the number of nanostrips per period for both the single-layer and double-layer absorbers in Figure 3g. The corresponding *FF* values are also provided in Table 2. Notably, the optimal *FF* increases as *N* increases and eventually reaches a plateau. This observation suggests that there exists an optimal number of resonators to integrate within the same period, given that each resonator possesses a finite absorption cross-section [22]. As depicted in Figure 3a, the use of a single-sized nanostrip in one period of a double-layer absorber yields two resonant wavelengths in the TM absorption spectrum, resulting in an *FF* of 68.3%. In contrast, the maximum *FF* achieved by the single-layer absorber with a single-sized nanostrip is only 37%. When *N* equals 5 in a period, the optimized *FF* and polarization extinction ratios are 87.2% and 38.51 for the double-layer absorber, and 71.0% and 30.8 for the single-layer absorber. This illustrates that the double-layer absorber can attain a higher TM absorption rate compared to the single-layer absorber, translating to lower *NETD* and *NeDoLP* values when integrated with a microbolometer.

**Figure 4:**
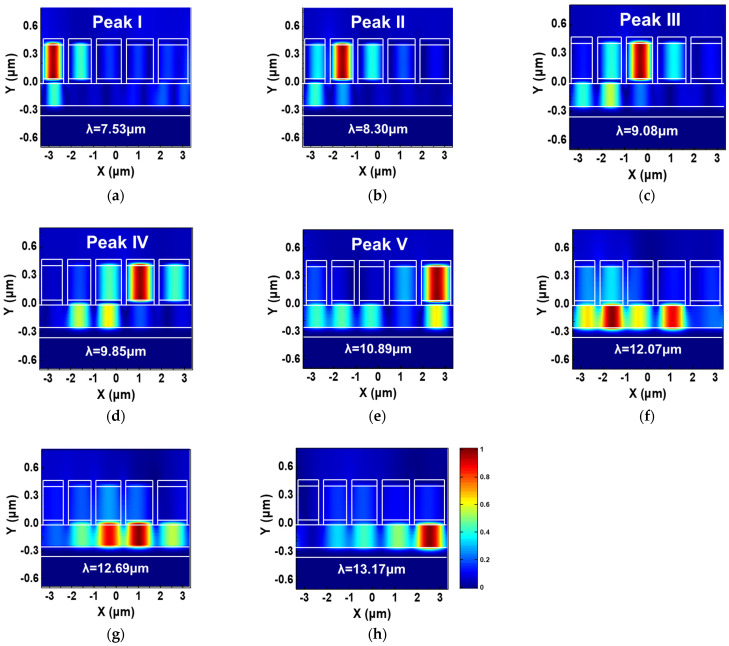
The distribution of the normalized magnetic field magnitude |H| in the optimized structure under TM polarization at (**a**) peak I (**b**) peak II (**c**) peak III (**d**) peak IV and (**e**) peak V of the spectral absorption shown in Figure 2. The distribution of |H| under TM polarization at (**f**) λ = 12.07μm (**g**) λ = 12.69μm and (**h**) λ = 13.17μm is also shown.

**Figure A1:**
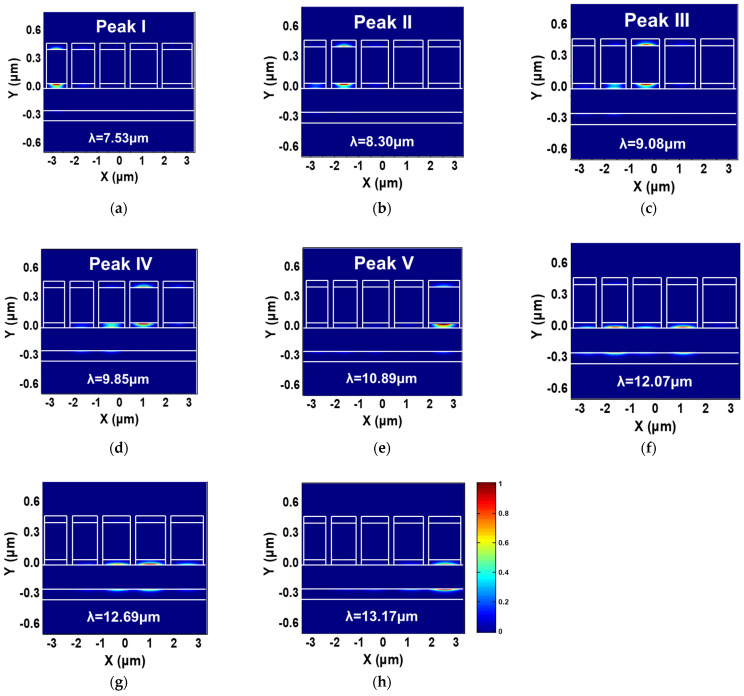
The distribution of the normalized absorption intensity in the optimized structure under TM polarization at (**a**) peak I (**b**) peak II (**c**) peak III (**d**) peak IV and (**e**) peak V of the spectral absorption shown in Figure 2. The absorption intensity distribution in the X–Y plane under TM polarization at (**f**) λ = 12.07μm (**g**) λ = 12.69μm and (**h**) λ = 13.17μm is also shown.

To gain deeper insights into the underlying physical mechanism behind the broadband absorption observed in Figure 2, we plot in Figure 4a–h the local distribution of magnetic field intensity at eight specific wavelengths: 7.53 μm (peak I), 8.3 μm (peak II), 9.08 μm (peak III), 9.85 μm (peak IV), 10.89 μm (peak V), 12.07 μm, 12.69 μm, and 13.17 μm (refer to Figure A1 in Appendix A for the corresponding power dissipation density). It is evident that at the first five resonant wavelengths, the magnetic fields primarily localize within specific discrete spacer regions situated between the second-layer nanostrip and the corresponding first-layer nanostrip, while the local fields elsewhere in the unit cell remain relatively weak. This localized electromagnetic field distribution suggests that the excitation of magnetic resonances (MRs) is the driving force behind the pronounced resonant absorption [32]. The broadband absorption is achieved by synergistically combining the resonances of multiple distinct resonators within the same unit cell [22]. Moreover, as the working wavelength progressively increases from 12 μm to 13.5 μm, the concentrated local magnetic fields shift from the discrete dielectric layer to the continuous spacer layer beneath it. Furthermore, the local magnetic fields cease to be concentrated under a single nanostrip and instead become focused beneath multiple adjacent nanostrips. This observation underscores the coupling between adjacent nanostrips in the horizontal direction.

**Figure 5:**
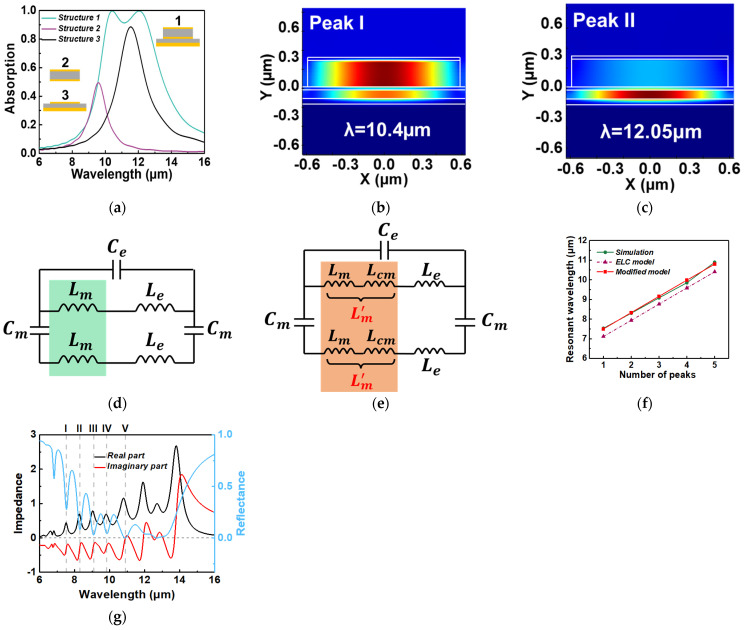
(**a**) The TM spectral absorption for different structures. The magnetic field magnitude |H| distribution in the X–Y plane at (**b**) peak I and (**c**) peak II for structure 1. (**d**) The equivalent LC circuit model for the fundamental MP mode. (**e**) The modified equivalent LC circuit model for the coupled MP mode. (**f**) Comparison of the resonant wavelength as a function of its serial number obtained by numerical simulation, ELC model, and modified circuit model, respectively. (**g**) Numerical calculation of the optical impedance (black and red solid line) and reflectance (blue solid line) of the optimized absorber under normal incidence.

The equivalent LC (ELC) circuit model has been demonstrated as an effective approach for predicting the fundamental magnetic resonance condition in nanostructures with MIM configuration [32,33,34,35]. In our case, we construct an LC circuit model to elucidate and forecast the resonant wavelengths of the MIMIM structure, as depicted in Figure 5d. The mutual inductance between the two parallel metal plates, separated by a dielectric spacer, can be represented as $L_{m}=0.5\cdot \mu_{0}\cdot W\cdot H$, where *H* signifies the spacer’s thickness, and $\mu_{0}$ denotes the vacuum permeability. *L_e_* represents the kinetic inductance, arising from the kinetic energy of electrons drifting within the nanoscale metallic strips. It can be calculated as $L_{e}=W/(\varepsilon_{0}\cdot \omega_{p}^{2}\cdot \delta_{Au})$, where $\varepsilon_{0}$ represents the permittivity of free space, and $\omega_{p}=1.367\times 10^{16}\;\mathrm{rad}/s$ corresponds to the plasma frequency of gold [35]. The power penetration depth, $\delta_{Au}$, is calculated as λ/4πκ, where κ denotes the extinction coefficient of Au. Calculations indicate that 13 nm < $\delta_{Au}$ < 14 nm, and for simplicity, we employ $\delta_{Au}$ = 13.7 nm. The capacitance between the nanostrips and backplane is represented as $C_{m}=c_{1}\cdot \varepsilon_{0}\cdot \varepsilon_{d}\cdot W/H$, where $\varepsilon_{d}=3.422$ denotes the relative permittivity of silicon [30], and *c*_1_ is a numerical factor accounting for the non-uniform charge distribution of the nanoscale capacitor [36]. Typically, 0.2 ≤ *c*_1_ ≤ 0.3, and in our calculations, we set *c*_1_ to 0.224, following [32] and [33]. Additionally, the capacitance *C_e_*, which considers the air gap between the top neighboring gold nanostrips, is defined as $C_{e}=\varepsilon_{0}\cdot H/\left(P-W\right)$, where *P* represents the period of the unit cell. For multi-sized nanostrips, $P=W_{i}+S$. The total impedance of the LC circuit shown in Figure 5d can be expressed as follows:

(9) $$Z_{tot}=\frac{i\omega \left(L_{m}+L_{e}\right)}{1-\omega^{2}C_{e}\left(L_{m}+L_{e}\right)}-\frac{2i}{\omega C_{m}}+i\omega \left(L_{m}+L_{e}\right)$$

where ω denotes the angular frequency. The prediction of the magnetic resonance condition can be determined by setting the total impedance $Z_{tot}$ to zero, which can be expressed as follows:

(10) $$\omega_{R}={\left(\frac{C_{m}+C_{e}-\sqrt{C_{m}^{2}+C_{e}^{2}}}{\left(L_{m}+L_{e}\right)C_{m}C_{e}}\right)}^{1/2}$$

We first use the ELC circuit model to analyze the simplest bilayer absorber structure with uniform nanostrip dimensions, and its structural parameters are shown in Table 2. As presented in Figure 5a, the green line corresponds to the absorption spectrum of the MIMIM structure (structure 1), identical to that in Figure 3a. The purple line and the black lines represent the absorption spectra of the upper three layers (structure 2) and the lower three layers (structure 3), respectively. It is important to note that the three insets in Figure 5a depict a single unit cell of the periodic structure, with the incident electric field oriented perpendicular to the nanostrips (TM polarization). The absorption peak of structure 2 does not exceed 50%, consistent with the prior literature [37]. The numerically simulated resonant wavelengths for structure 2 and structure 3 are found to be 9.58 μm and 11.54 μm, respectively. Employing the LC circuit model, the predicted resonant wavelengths for structure 2 and structure 3 are 9.55 μm and 11.56 μm, respectively, closely matching the numerical results. However, for the absorption peak of structure 1, the ELC circuit model (as shown in Figure 5d) encounters challenges. This is due to the significant coupling between the magnetic resonant fields within the first spacer and the second spacer of the bilayer absorber structure, as evident from the magnetic field distribution of resonance peaks I (Figure 5b) and II (Figure 5c). A previous study [38] successfully predicted the absorption spectrum of stacked double-ring resonators based on the equivalent circuit model and coupled lumped-element LC resonators. However, this approach involves a complex calculation process and requires the fitting of multiple parameters to match simulation results. Here, we propose a simpler modified method. By observing the magnetic field distribution in Figure 5b,c, we can find that the coupling occurs between the MP in the first spacer and the second spacer. Initially, we assume that the capacitance term in the ELC circuit model and the inductance *L_e_*, resulting from the drifting charge, remain unchanged.

Subsequently, we introduced two additional coupling mutual inductances *L_cm_* into the circuit due to the mutual inductance effect between two MPs, as illustrated in Figure 5e. We define $L_{m’}=L_{m}+L_{cm}=\gamma_{c}*L_{m}$, where $\gamma_{c}$ > 1 is referred to the coupling coefficient. It is important to note that $\gamma_{c}$ can vary for different resonance wavelengths. By replacing *L_m_* in Equation (10) with *L_m_*’, we were able to calculate the resonant wavelengths. For resonant peaks I and II labeled in Figure 5a, the values of *γ_c_* are *γ_c_*_1_ = 1.24 and *γ_c_*_2_ = 1.16, and the two resonant wavelengths calculated according to the modified circuit model are 12.04 μm and 10.4 μm, which are very close to 12.05 μm and 10.4 μm obtained by numerical simulation. Also, *γ_c_*_1_ > *γ_c_*_2_ indicates that the coupling of resonant peak I is stronger than that of resonant peak II, which is consistent with the magnetic field distributions in Figure 5b,c. Note that the modified LC model is based on several approximations and does not account for the complex interaction between multi-MPs and other modes. Here, our modified model can successfully analyze the first five resonant peaks identified in Figure 2, but it fails to predict the resonant peaks from 12 μm to 13.5 μm because these peaks are coupled together and cannot be distinguished. In Figure 5f, the green line represents the resonant wavelengths obtained by numerical simulation, and the purple dotted line represents the resonant wavelengths calculated by the conventional LC circuit model (Figure 5d). It can be seen that there is an offset between these two lines, and by using our modified model, this offset can be easily eliminated. The RMS error between the resonant wavelengths predicted by our model (red solid line) and the numerically simulated resonant wavelengths is <3.5%. Among them, the coupling coefficients *γ_c_* of the five resonant peaks are 1.13, 1.12, 1.11, 1.1, and 1.09, respectively, which decrease linearly.

To further reveal the underlying mechanism of the broadband absorption of the optimized structure, we consider an optical impedance matching picture—by matching the optical impedance *Z* to the value of the incident medium, a zero reflectance can be obtained [34]. The impedance of our optimized absorber can be extracted from the scattering parameter of the numerical simulations [35] using

(11) $$Z=\sqrt{\frac{{\left(1+S_{11}\right)}^{2}-S_{21}^{2}}{{\left(1-S_{11}\right)}^{2}-S_{21}^{2}}},$$

where *S*_11_ and *S*_21_ represent the scattering matrix coefficients for the reflection and transmission of normal incident light with TM polarization, respectively; due to the presence of the metal backplate, the transmission of the incident light is blocked, and *S*_21_ is set to zero. Figure 5g compares the simulated results for the normalized real and imaginary parts of the optical impedance with the reflectance of the optimized absorber. It is seen that the real and imaginary parts of the optical impedance *Z* approach the respective vacuum values of one and zero at multiple wavelengths, resulting in multiple minima in the spectral reflection and efficient broadband absorption.

The current configuration of the focal plane polarimeter involves resolving the polarization states of incoming light through the placement of a micro-polarizer array (MPA) in front of the focal plane array. However, this transmissive architecture unavoidably introduces optical crosstalk due to factors like diffraction and oblique incidence. This optical crosstalk can significantly impair the performance of polarization selection [6,36]. In contrast, plasmonic metamaterial absorbers harness localized surface plasmon resonances (LSPRs), and their absorption characteristics remain largely unaffected by the angle of incidence of light. Furthermore, these absorbers are directly integrated with the sensing layer of the detector’s pixels, ensuring robust polarization selection performance to a great extent.

**Figure 6:**
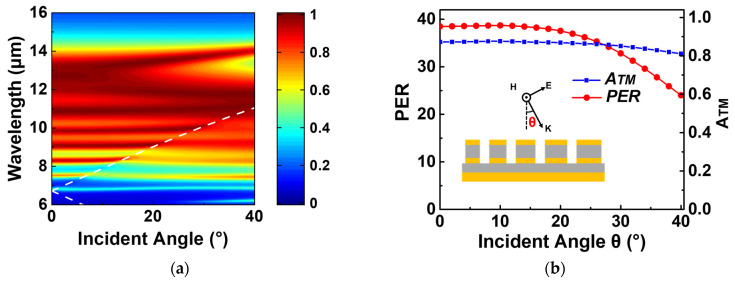
(**a**) Spectral absorption contour plot of the optimized MIMIM absorber at different incident angles for TM polarization. The white dashed line represents the resonant wavelength of the surface plasmon polariton as a function of the incident angle. (**b**) The polarization extinction ratio and the TM spectral absorption as a function of incident angle.

In a typical IR camera, the detector’s pixels are situated at the focal plane where the light converges. Thus, the integrated absorbers must maintain a wide acceptance cone. For instance, assuming an optical system with an f-number of F/#1, the incoming light forms a ray cone with a close 30-degree half angle. Figure 6a illustrates the TM spectral absorption of the optimized absorber as a function of the incident angle. The double-layer absorber exhibits absorption characteristics that remain stable over a range of angles, similar to the single-layer version [8]. It is observed that with increasing incidence angle, the excited SPPs degrade the LSPRs, resulting in a deterioration of the absorption rate, as indicated by the white dotted line in Figure 6a.

Figure 6b presents the average absorption of TM polarization as a function of the incident angle (blue line), demonstrating that the optimized double-layer absorber maintains high absorption within a 30-degree angle range. Compared to the case of normal incidence, the absorption at angles up to 30 degrees decreases by only 2.374%, with the effect of SPPs being nearly negligible. It is worth noting that maintaining an efficient polarization extinction ratio under oblique incidence is challenging for the MPA-based transmissive architecture [37]. The red solid line in Figure 6b reveals that at an incident angle of 30 degrees, the absorber’s polarization extinction ratio decreases from 38.51 (at normal incidence) to 32.84. In other words, the *PER* decreases by only 15% due to an increased absorption of TE polarization.

**Figure 7:**
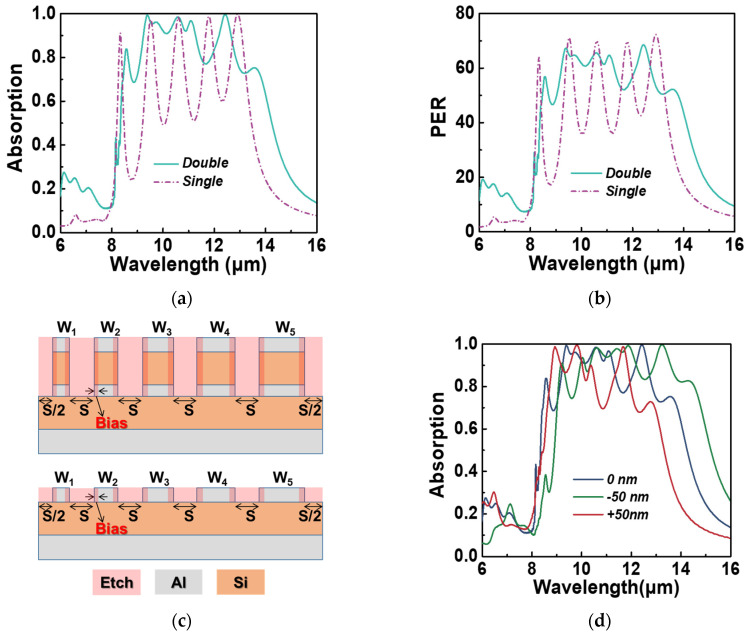
(**a**) The TM spectral absorption and (**b**) the corresponding extinction ratio of the optimized single-layer CCA (purple dotted line) and double-layer CCA (solid green line) under normal incident light. (**c**) Schematic diagram of fabrication errors for two types of CCA. (**d**) The TM spectral absorption of optimized double-layer CCA at different fabrication errors.

While noble metals like gold and silver have been utilized as plasmonic materials in various applications such as biosensors [39] and metamaterial absorbers [23], they are not compatible with the silicon IC (integrated circuit) process. Here, we explore the use of aluminum as the plasmonic material for creating metamaterial absorbers due to its abundance, cost-effectiveness, and compatibility with the silicon IC process. In conventional I-line steppers, the minimum feature size of generated patterns typically ranges from 0.3 μm or larger [40], while the critical size (CD, specifically, the *S*) of all the optimized absorbers mentioned earlier is larger than 300 nm. To ensure that the optimized patterns can be fabricated using I-line steppers, we manually set the range of *S* to be above 400 nm during the optimization process. For the sake of convenience, we refer to the optimized aluminum-based absorber structures as CMOS-compatible absorbers, abbreviated as CCAs. Table 3 provides the parameters of the optimized CCAs. Figure 7a and Figure 7b depict the TM spectral absorption and polarization extinction ratio of the double-layer (solid green line) and single-layer (purple dotted line) CCAs, respectively. In the case of the single-layer CCA, the optimal *FF* and *PER* are 63.4% and 45.53, respectively. In contrast, for the double-layer CCA, the optimal FF and polarization extinction ratio are 81.6% and 55.47, respectively.

To assess the fabrication tolerance of the CMOS-compatible absorber (CCA), we examine the TM spectral absorption of the double-layer CCA when subjected to ±50 nm fabrication errors. It is important to note that a ±50 nm fabrication error corresponds to a variation in the width of the nanostrip by 100 nm, which constitutes 25% of the critical size (CD) of 400 nm. As depicted in Figure 7c, the CCA can be realized through patterning and etching into the multilayer film, with the width of the etched region denoted as S. The term “Bias” represents the fabrication errors introduced during the etching process. A positive value implies that the width W becomes smaller, while a negative value indicates that W becomes larger. Analyzing the calculated TM spectral absorption of the double-layer CCA presented in Figure 7d, we observe that the CCA maintains high-broadband TM polarization absorption even in the presence of fabrication errors. Although there are slight alterations in the center wavelength and bandwidth of the high absorption band, the CCA exhibits a considerable level of fabrication tolerance.

**Figure 8:**
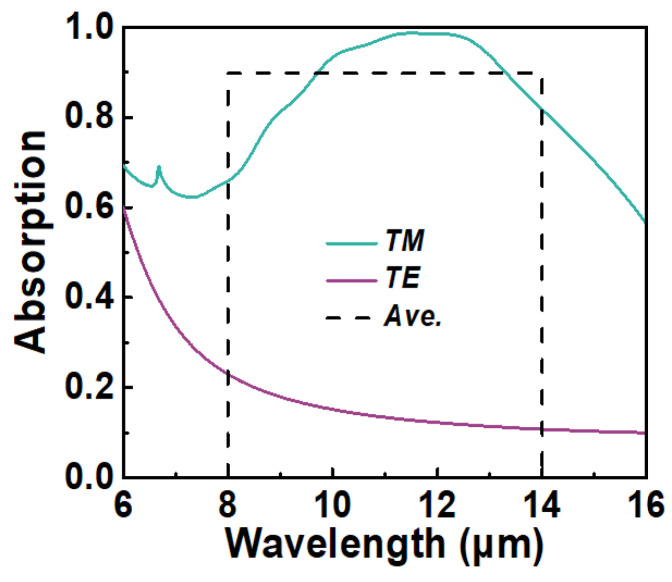
The spectral absorption of TE polarization and TM polarization for optimized Ti-based absorber at normal incidence.

In addition to aluminum, titanium is also a commonly used CMOS-compatible plasmonic material. Many works found in the literature have reported the use of highly lossy plasmonic materials such as titanium to achieve broadband absorption [40,41]. Thus, we also conducted PSO optimization on the MIMIM structure by setting the metals as titanium. The optimization results plotted in Figure 8 show that the average TM absorption of the optimized absorber can reach 90%, but the PER is only 6.236, which is too low for efficient polarization detection. The primary reason for the low polarization extinction ratio in this case is that highly lossy metals like titanium have a lower carrier concentration, leading to a smaller magnitude of permittivity in the infrared range compared to highly conductive noble metals like gold, silver, and aluminum. Consequently, these high-loss metals are less effective at reflecting incident TE-polarized light, resulting in higher TE absorption. This implies that, for polarization detection schemes, integrated MIM absorbers should employ metals with high conductivity, such as gold, silver, or aluminum, rather than highly lossy metals like titanium, chromium, or nickel, as the latter may not provide sufficient polarization selectivity.

To maximize the polarization selection performance, i.e., the *NeDoLP*, the *NETD* should be minimized. However, there is a trade-off between minimizing the *NETD* and achieving a suitable time constant $\tau_{th}$, which is another critical parameter of the thermal detector. To account for this trade-off and provide a comprehensive measure of a thermal detector’s performance, a figure of merit (*FOM*) has been defined for conventional microbolometers [42]:

(12) $$FOM=NETD\times \tau_{th}=\frac{4C_{th}V_{niose}}{TCR\cdot FF\cdot \varepsilon \cdot V_{bias}\cdot A_{D}\cdot {\left(\Delta P/\Delta T\right)}_{\lambda_{1}-\lambda_{2}}}=\frac{C_{th}}{\varepsilon }\times \frac{4V_{niose}}{TCR\cdot FF\cdot V_{bias}\cdot A_{D}\cdot {\left(\Delta P/\Delta T\right)}_{\lambda_{1}-\lambda_{2}}}$$

In this new FOM, a smaller value indicates better polarization detection performance. For the polarization-selective microbolometers mentioned in this work, ε in the formula should be replaced with the average spectral absorption *A_TM_*. And $C_{th}=C_{bolo}+C_{MA}$ is equal to the sum of the heat capacity of the bolometer *C_bolo_* and the heat capacity of the integrated metamaterial absorber *C_MA_*. It can be seen from Equation (12) that the ratio of total heat capacity *C_th_* to ε (replaced by *A_TM_*) affects the *FOM* in such a way that a larger ratio (*C_th_/A_TM_*) increases the *FOM* (assuming everything else is constant). Note that here, we ignore the spectral selectivity of *A_TM_*, but this has little effect on the conclusion. According to Equation (7), the *NeDoLP* is proportional to the *NETD*. So *NeDoLP* and $\tau_{th}$ also have a trade-off. Thus, we define another *FOM*, which is also proportional to the ratio of the total heat capacity to the TM absorption:

(13) $$\;FOM_{P}=\left(NeDoLP\times \tau_{th}\right)\propto (C_{bolo}+C_{MA})/A_{TM}$$

**Figure 9:**
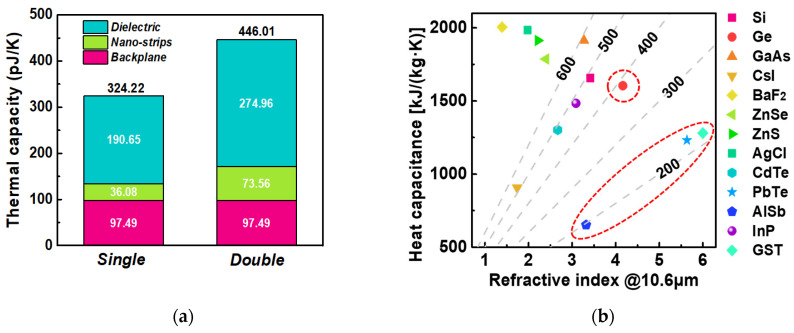
(**a**) The heat capacity contribution of each part of single-layer and double-layer CCAs. (**b**) The volume-specific heat capacity and infrared refractive index (10.6 μm) of some alternative infrared-transparent materials. The red dotted coils in the picture show some potential material choices for the spacer.

The newly defined *FOM_p_* can be used to comprehensively evaluate the performance of a polarization-selective thermal detector. To more intuitively compare the performance of single-layer and double-layer absorbers in real devices, we selected the two CCAs shown in Figure 7a. Assuming that the area size of the microbolometer and the integrated absorber is 20 μm × 20 μm, then the heat capacity of the membrane is 350 pJ/K, as recalculated from [42]. The total heat capacity for single-layer CCA and double-layer CCA is calculated as 324.2 pJ/K and 446.0 pJ/K, respectively, as shown in Figure 9a.

To simplify thermal analysis, the specific heat capacity and density can be treated as bulk material values. The physical parameters used for thermal capacity calculations are detailed in Table 4 [43,44,45]. Combining the average absorption given in Table 3, we can compare the *FOM_p_* of the two structures by comparing the ratio of the total heat capacity to the average absorption. Calculation results reveal that the double-layer CCA scheme has a *FOM_p_* about 10% smaller than the single-layer CCA scheme. This signifies that the proposed double-layer CCA scheme indeed achieves higher overall performance. To further enhance the overall performance, we need to explore methods to reduce the heat capacity of the absorber while increasing absorption rates. One way to reduce the heat capacity is to decrease the thickness of the metal layers. Currently, the metal backplate has a thickness of 100 nm, which greatly exceeds the skin depth in the infrared spectrum, allowing room for thickness reduction. Likewise, the thickness of the metal nanostrips can also be reduced. Another way is to reduce the heat capacity of the dielectric spacer, because it contributes to more than half of the absorber’s total heat capacity, as illustrated in Figure 9a. This can be achieved by replacing silicon with dielectric materials with a small volumetric heat capacity and a sufficiently high refractive index [11]. Figure 9b displays the volume-specific heat capacity and infrared refractive index (at 10.6 μm) of several infrared-transparent materials [46,47,48,49,50]. A smaller ratio of the dielectric material’s specific heat capacity to its refractive index results in an improved overall performance of polarization detection. Based on the data presented in Figure 9b, potential material choices for the spacer include Ge, AlSb [51], GST [52,53], and PbTe [54].

## 3. Conclusions

It is a promising way to realize uncooled infrared polarization detection by integrating metamaterial absorbers. However, the polarization-selective metamaterial absorbers reported so far have the problem of insufficient polarization absorption and extinction ratio. Because of this problem, this paper combines the advantages of horizontal integration and vertical integration to propose a hybrid integrated multi-sized multilayered nanostrip structure as the design framework of this metamaterial absorber. Through a comprehensive analysis of NeDoLP-based polarization imaging performance and the interplay between polarization absorption rates and polarization extinction ratios, we have concluded that enhancing the absorption rate of TM polarization is more important. Guided by this, combined with a custom-developed PSO algorithm, we optimized a multilayer design that achieves an average polarization-selective absorption rate of 87.2% in the entire LWIR band and an excellent PER of up to 38.51, and compared it with the optimized MIM structure of the single dielectric layer. We used a modified ELC model and optical impedance analysis methods to reveal the physical mechanism by which the optimal structure has broadband polarization-selective high absorption. We have also investigated the absorption characteristics concerning incident angles for the optimized structure.

To address material compatibility and cost considerations in the silicon IC process, we opted to replace the noble metal gold with aluminum. Subsequently, we re-optimized the design and conducted a comparison with the single-layer MIM structure. Our analysis also delved into the impact of etching errors introduced during the etching process. The outcomes of this investigation revealed that the proposed structure exhibited a considerable degree of fabrication tolerance. Furthermore, we explored the effects of substituting the material with titanium (Ti). In doing so, we observed that the high polarization-selective absorption characteristics were no longer present, and the polarization extinction ratio (PER) dropped to a mere 6.236. This PER value proved insufficient to achieve high-performance polarization detection.

To address the question of whether the multi-sized bilayer absorber, despite improving the absorption rate, also increases the thickness (and subsequently, heat capacity), and whether it enhances the overall performance of uncooled infrared polarization detection compared to the traditional single-layer MIM absorber, we have introduced a novel figure of merit (FoM). This FoM provides a visual means to compare the contributions of different polarization-selective absorbers to the performance of infrared polarization detection. Our findings indicate that the optimal bilayer absorber surpasses the single-layer absorber in terms of performance according to the FoM. Furthermore, the FoM offers insights into avenues for further improving overall performance, including strategies such as reducing the thickness of the metal layer and selecting a dielectric material with a higher volume-specific heat capacity-to-refractive index ratio.

## Figures and Tables

**Table 1:** Comparison between our work and the previously reported absorbers with broadband absorption and polarization selectivity in the LWIR band.

Source	Average Absorbance (TM)	FWHM (μm)	Center Wavelength (μm)	Quality Factor	Extinction Ratio	Thickness (nm)	Integration Method
This Work	87.2%@8–14 μm	6.37	11	1.73	38.51	828	Hybrid
[16]	55.6%@7–13 μm	3.08	9.84	3.2	~20	456.5	Horizontal
[12]	77.3%@8–12 μm	3.94	10	2.54	37	350	Horizontal
[17]	90%@8–12 μm	4.62	10	2.17	Low	1560	Horizontal
[18]	>95%@2–12	>9.76	6.86	<0.7	~30	>8080	Vertical

**Table 2:** Relevant parameters of the optimized single-layer and double-layer absorbers with different numbers of nanostrips per period.

Double-Layer Absorbers (*H*_1_ = 100 nm, *H*_3_ = *H*_5_ = 50 nm)						
*N*	*S* (nm)	Δ (nm)	*H*_2_ (nm)	*W* (nm)	*H*_4_ (nm)	*FF* (%)
1	104.1	55.6	77.6	1160.8	249.5	68.3
2	227.9	181.4	123.5	1208.2	313.8	75.3
3	198.2	177.5	133.5	1163.2	307.9	83.2
4	277.9	123.9	236.5	1184.1	431.0	85.2
5	233.3	105.3	242.3	1111.9	385.8	87.2
**Single-layer absorbers (*H*_1_ = 100 nm, *H*_3_ = 50 nm)**						
1	106.0	125.9	111.1	1116.0	N/A	37.3
2	287.0	195.2	195.3	1185.0	N/A	50.8
3	299.9	199.6	239.9	1153.7	N/A	60.9
4	256.3	194.5	237.3	1079.8	N/A	67.1
5	299.4	160.6	283.8	1058.9	N/A	71.0

**Table 3:** Relevant parameters of the optimized single-layer and double-layer CCAs.

Double-Layer CCA						
N	S (nm)	Δ (nm)	H_2_ (nm)	W (nm)	H_4_ (nm)	FF (%)
5	400.0	198.6	97.9	1228.6	420.5	81.6
**Single-layer CCA**						
*N*	*S* (nm)	Δ (nm)	*H_2_* (nm)	*W* (nm)	*H_4_* (nm)	*FF* (%)
5	400.4	169.5	287.7	1140.3	N/A	63.4

**Table 4:** Physical properties of materials for thermal analysis.

Materials	Thermal Conductivity (W/m·K)	Specific Heat Capacity (J/kg·K)	Volumetric Heat Capacity (J/cm^3^·K)	Density (kg/m^3^)
Au	316	129	2.490	19,300
Al	236	902	2.437	2702
Si	148	711	1.657	2330

## Data Availability

Data are contained within the article.
